# Tryptophan‐kynurenine metabolism during acute alcohol withdrawal in patients with alcohol use disorder: The role of immune activation

**DOI:** 10.1111/acer.14920

**Published:** 2022-08-20

**Authors:** Sergei Mechtcheriakov, Gabriele V. Gleissenthall, Simon Geisler, Kathrin Arnhard, Herbert Oberacher, Timo Schurr, Georg Kemmler, Christine Unterberger, Dietmar Fuchs

**Affiliations:** ^1^ University Clinic of Psychiatry I, Department of Psychiatry, Psychotherapy, Psychosomatics, and Medical Psychology Medical University of Innsbruck Innsbruck Austria; ^2^ Institute of Biological Chemistry, Biocenter Medical University of Innsbruck Innsbruck Austria; ^3^ Institute of Legal Medicine and Core Facility Metabolomics Medical University of Innsbruck Innsbruck Austria

**Keywords:** alcohol use disorder, immune activation, tryptophan‐kynurenine metabolism

## Abstract

**Background:**

Recent research has suggested that excessive alcohol consumption in patients with alcohol use disorder (AUD) is associated with chronic immune activation, which affects the metabolism of the neurotransmitter precursor amino acid tryptophan (TRP) and contributes to the complex pathophysiology of AUD. Our study investigated possible immune‐associated alterations of TRP to kynurenine (KYN) metabolism in patients with AUD during acute alcohol withdrawal.

**Methods:**

We measured serum concentrations of TRP, KYN, quinolinic (QUIN), kynurenic acid (KYNA), and the immune activation marker neopterin (NEO) at the first, fifth and 10th day of alcohol withdrawal in patients with AUD, who attended a standardized in‐patient treatment program and underwent a detailed clinical assessment.

**Results:**

Data from these individuals were compared to data from a reference control group (RCG). The primary outcome measures were the differences in serum concentrations of metabolites between AUD patients and RCG and correlations between NEO and metabolites of the tryptophan‐kynurenine pathway. *r* = 0.695, *p* < 0.001) in the AUD group. Mixed models analysis showed that NEO concentrations were positively associated with QUIN but not with KYNA concentrations. Several behavioral symptoms correlated positively with QUIN concentrations and negatively with the KYNA/QUIN ratio.

**Conclusions:**

Our findings demonstrate that the changes in TRP catabolism in acute alcohol withdrawal resulting in increased KYN production could reflect the involvement of immune‐associated activation of the enzyme indoleamine 2,3‐dioxygenase, as NEO concentrations correlated with the KYN/TRP ratio. In addition, our data show that this low‐grade immune activation may cause an imbalance in the production of neurotoxic and neuroprotective kynurenine metabolites in AUD.

## Graphical abstract

We studied patients with AUD during acute withdrawal and showed that alcohol‐induced low‐grade immune activation is associated with a stimulation of the kynurenine route of tryptophan metabolism. These changes are indicated by the increase of the kynurenine‐to‐tryptophan ratio and by the positive correlation of this ratio with neopterin concentrations. Furthermore, the immune activation seems to cause a shift in catabolism of the kynurenine away from the neuroprotective kynurine acid towards neurotoxic metabolites such as quinoline acid.

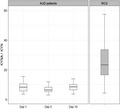

## INTRODUCTION

There is an ample body of evidence that alcohol use disorder (AUD), a chronic psychiatric disease with numerous somatic manifestations, is associated with chronic activation of the immune system (González‐Reimers et al., 2014; Kelley & Dantzer, 2011). One of the important effects of the chronic immune activation in AUD is the disturbance of the tryptophan (TRP) metabolism (Gleissenthall et al., 2014; Leclercq et al., 2021). Because of its susceptibility to modulation by immune‐mediated effects and its involvement in functionality of several neurotransmitter systems, the TRP metabolism has recently attracted considerable research attention in different diseases (Capuron & Miller, 2011; De Picker et al., 2020; Milaneschi et al., 2021; Morris et al., 2016; Oxenkrug, 2010). As precursor of serotonin, TRP represents one of the possible links between immunological phenomena and neurotransmitter metabolism (Christmas et al., 2011; Maes et al., 2011). Furthermore, chronic immune activation and immune‐triggered alterations of TRP metabolism have been reported to be associated with behavioral and affective symptoms in different clinical conditions (Capuron et al., 2011; Hüfner et al., 2020; Myint & Kim, 2014; O'Farell & Harkin, 2017; Schwarcz & Stone, 2017).

Generally, TRP is degraded to kynurenine (KYN) by the combined activity of the mainly hepatic enzyme tryptophan 2,3‐dioxygenase (TDO) and the two extrahepatic isoforms of indoleamine 2,3‐dioxygenase (IDO) 1 and 2. Under normal conditions, the IDO‐1 degrades approximately 5% to 15% of TRP but it can be considerably activated by cytokines (Schröcksnadel et al., 2006). IDO‐2 is an isoenzyme with different expression pattern, kinetic activity and substrate range compared to IDO‐1 (Ball et al., 2007). Generally, the TDO is the rate limiting enzyme which covers about 85% to 95% of TRP degradation to KYN (Badawy, 2013; Bender, 1983).

Excessive alcohol consumption in AUD affects TRP metabolism by different mechanisms (Badawy et al., 1998; Badawy, 2002; Badawy et al., 2009; Leclercq et al., 2021) including stress‐related activation of TDO. In contrast, the role of IDO in this context is still to be clarified, although recent data have shown a moderate IDO‐activation in the mid‐term alcohol withdrawal (Gleissenthall et al., 2014) and increased activity of TDO and IDO in acute alcohol withdrawal in AUD patients (Leclercq et al., 2021).

Recent studies have also suggested the importance of the downstream KYN catabolites such as quinolinic acid (QUIN) and kynurenic acid (KYNA) since these catabolites themselves possess neuroactive properties (Myint & Kim, 2014; Vécsei et al., 2013). KYNA possesses potentially neuroprotective anti‐glutamatergic properties (Urbańska et al., 2014), while QUIN exerts neurotoxic effects by several mechanisms including glutamatergic agonism (Guillemin, 2012; Schwarcz & Du, 1991).

The present study focuses on the interactions between immune system and TRP‐KYN metabolism during the acute alcohol withdrawal in patients with severe AUD. To this end, we studied the relationships between serum concentrations of neopterin (NEO), which is known as a marker of cellular immune activation (Gostner et al., 2020; Murr et al., 2002), and an amino acid TRP and its metabolites KYN, QUIN and KYNA during the first 10 days of alcohol withdrawal in AUD patients in comparison to a reference group of controls.

## METHODS

### Patients with AUD and treatment regime

Twenty two consecutive in‐patients, who were admitted at the University Clinic of Psychiatry I of Medical University Innsbruck for treatment of acute alcohol withdrawal, were recruited for this study. The patients underwent a standardized in‐patient alcohol withdrawal treatment based on symptom‐guided benzodiazepine treatment with oxazepam. Prior to inclusion in the study all patients were in detail informed about the study participation terms and signed the informed consent according to the study protocol approved by the Ethics Committee of Medical University of Innsbruck (EC Protocols AN2014‐0134 336/4.15 and AN‐2014‐0018).

### Inclusion criteria

The following inclusion criteria have been applied in this study: (1) at least 2 years of existing alcohol dependence or AUD as diagnosed according to the International Classification of Diseases (ICD‐10) or DSM IV respectively; (2) average daily alcohol consumption above 60 g/day; (3) age between 18 and 65 years; (4) motivation for an abstinence‐oriented treatment; (5) informed consent.

### Exclusion criteria

Patients with acute or exacerbated severe psychiatric disorders other than AUD or severe cognitive impairment, which precluded participation in the post‐withdrawal treatment program as well as known chronic severe immune disease, severe alcohol‐induced hepatitis, cancer or pregnancy were not included in this study.

### Study design

Clinical investigations and ratings as well as acquisition of blood samples were performed three times during the treatment: on the first, fifth and 10th day after the admission.

### Acquisition of blood samples and biological assays

Blood samples were collected by trained clinic personnel between 6.00 a.m. and 10.00 a.m. and transported to the laboratory facility. Sera were stored at −20°C until thawed for measurements. TRP and KYN concentrations were determined by high‐performance liquid chromatography as described earlier (Laich et al., 2002; Widner et al., 1997). Natural fluorescence (286 nm excitation and 366 nm emission wavelengths) was used for TRP detection. KYN concentrations were measured by ultraviolet absorption at 360 nm. The ratio of KYN to TRP (KYN/TRP) concentrations was calculated as index of TRP breakdown (Fuchs et al., 1990). Concentrations of NEO were measured by enzyme‐linked immunosorbent assay (BRAHMS GmbH) with a detection limit of 2 nM (Mayersbach et al., 1994).

Additionally, the serum samples were analyzed with liquid chromatography–tandem mass spectrometry (LC–MS/MS) to quantify QUIN and KYNA (Arnhard et al., 2018). Deuterated analogues of KYNA, and QUIN served as internal standards (IS). Serum samples were prepared for LC–MS/MS analysis by protein precipitation with acetonitrile. 50 *μ*l serum were mixed with 5 *μ*l of internal standard solution (c(KYNA‐D5) = 200 ng/ml, c(QUIN‐D3) = 2.0 *μ*g/ml), 25 *μ*l of water and 70 *μ*l of acetonitrile. After sonication (5 min) and centrifugation (12,000 × g, 5 min), 100 *μ*l of the supernatant was mixed with 5 *μ*l ammonium hydroxide (25%). 5 *μ*l of protein precipitated serum were injected onto the chromatographic column (Luna NH2, 150 × 1.0 mm, 3 *μ*m, 100 Å, Phenomenex). Separation was accomplished with a 6 min gradient of 50% to 5% acetonitrile in aqueous ammonium acetate (5 mM, pH 9.5) at a flow rate of 35 *μ*l/min and detected on a quadrupole−quadrupole−time‐of‐flight instrument (TripleTOF5600+, Sciex) using electrospray ionization in negative ion mode. The applied acquisition strategy involves the collection of full scan MS/MS spectra and post‐acquisition extraction of fragment ion mass traces that are specific for the analytes and IS of interest. Serum treated with activated charcoal was used as surrogate matrix to prepare calibration standards (KYNA: 1 to 50 ng/ml, QUIN: 20 to 1000 ng/ml). The LC–MS/MS method was validated. The parameters tested for each compound included calibration model, limit of quantification, accuracy, reproducibility, sample stability, recovery, and matrix effects. Reference concentrations of KYN, TRP, NEO, KYNA and QUIN were acquired in the study of 100 healthy blood givers as reported previously (Arnhard et al., 2018; Geisler et al., 2015).

### Clinical assessments

The study‐related investigation procedures were embedded in a semi‐structured routine clinical psychiatric investigation. We used Alcohol Use Disorder Identification Test (AUDIT; Saunders et al., 1993) to capture the severity of alcohol dependence. Detailed data upon alcohol consumption in the last 3 months have been collected by means of Timeline Follow Back scale (TLFB; Sobell, 1992). In order to monitor withdrawal‐associated symptoms and to scan for other potential neuropsychiatric conditions the following clinical rating instruments were applied during the study: Day 1—Clinical Institute Withdrawal Assessment for Alcohol Scale (CIWA; Sullivan et al., 1989) and German version of Obssessive‐Compulsive Drinking Scale (OCDS‐g; Mann & Ackermann, 2000), day 5—CIWA and Beck Depression Inventory (BDI; Hautzinger, 2002) and day 10—CIWA and BDI. Liver function parameters (alanine aminotransferase—ALAT, aspartate aminotransferase—ASAT, gamma‐glutamyl transferase—GGT) as well as blood count and CRP were acquired routinely to assess the severity of clinical involvement.

### Statistics

The distribution of the biochemical parameters was checked for normality by means of the Shapiro–Wilk test and by visual inspection. As the majority of the parameters showed significant departures from a normal distribution, analysis of these parameters in the course of time was performed by the non‐parametric Friedman test. Differences between patients and controls were analyzed by Mann–Whitney‐*U* test. Associations between biochemical parameters were analyzed by means of Spearman rank‐correlation coefficients.

In order to detect possible differences in effects of immune‐activation on KYNA and QUIN an analysis by linear mixed models was performed, using a random intercept model and assuming an first‐order auto‐regressive correlation structure (AR1) for the factor time (day 1, 5, and 10). Thus, allowing to account for both, intra‐individual and inter‐individual variability of change in the estimation of model parameters. Two analyses were conducted, one for the dependent variable QUIN and the other one for KYNA, considering NEO and KYN concentrations as the independent variables. This analysis was also repeated for NEO and KYN/TRP as independent variables. To account for deviations from the normal distribution, a modified version of each analysis was performed using log‐transformed concentration values.

## RESULTS

### Patients' demographic and clinical characteristics

Twenty‐two consecutive in‐patients (16 men and six women; mean age: 43.5 year, SD: 10.6 year) completed this study. Mean total AUDIT score was 30 (SD: 7.2), total alcohol consumption in the last 30 days prior to admission was estimated as 5999 g of pure alcohol in average (SD: 3099 g) and in the last 90 days as 17,527 g alcohol in average (SD: 8835 g), which corresponded to the average of about 200 g alcohol daily. At the beginning of the study the patients were abstinent in average for 2.95 days (SD: 3.9 days).

All recruited patients developed a moderate to severe alcohol withdrawal that required treatment with benzodiazepines. At the beginning of the treatment, the average daily dose of oxazepam was 175 mg (SD: 83 mg). At the fifth day the oxazepam dose averaged by 117 mg/day (SD: 77 mg/day) and the 10th day—by 65 mg/day (SD: 49 mg/day).

### Routine laboratory parameters

As could be expected in this group of patients, the activities of several liver enzymes were elevated at admission and corresponded to the reported high consumption rates. The average values of GGT at admission were 199 U/L (SD: 285 U/L, reference values: 10 to 71 U/L); the ALAT was 65 U/L (SD: 72 U/L, reference values: 10 to 50 U/I) and the ASAT was 61 U/L (SD: 48 U/L, reference values: 10 to 50 U/I). The CRP value at admission was 0.93 mg/dl (SD: 2.04 mg/dl, reference values: <0.50 mg/dl).

### Tryptophan‐kynurenine metabolism and immune activation

**FIGURE 1:**
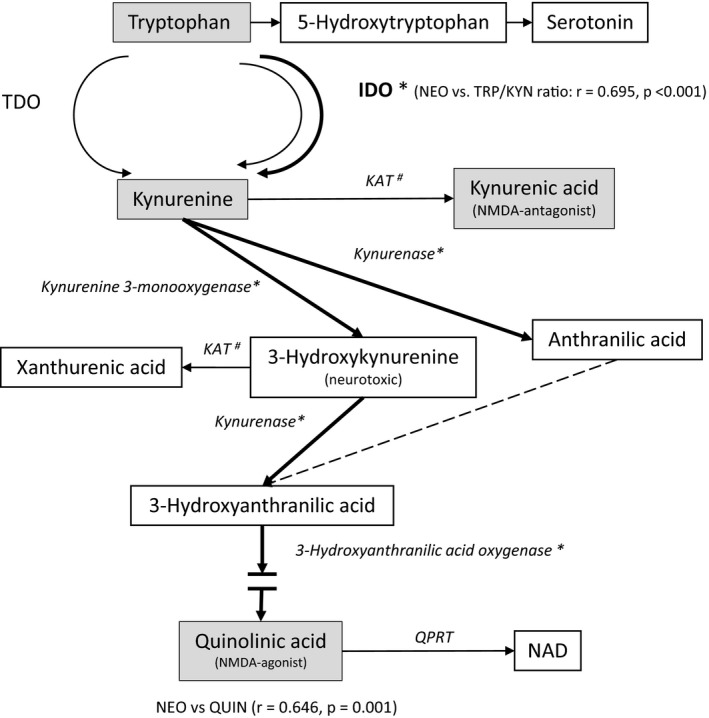
Tryptophan‐kynurenine pathway. *—induced by immune activation, ^#^—several KAT enzymes; QPRT, quinolinic acid phosphoribosyltransferase; KAT, kynurine aminotransferase; TDO, tryptophan deoxygenase; IDO, indolamine 2,3‐dioxigenase; NAD, nicotinamide adenine dinucleotide; KMO, kynurenine monooxygenase. The diagram presents schematically the tryptophan‐kynurenine pathway. Tryptophan is catabolized to kynurenine by TDO and IDO isoenzymes. IDO‐1 can be induced by immune activation, which is reflected by the correlation between neopterin and tryptophan to kynurenine ratio. Kynurenine is further catabolized along two main routes: either towards production of kynurenic acid by KAT enzymes or towards the production of 3‐hydroxykynurenine and consequently to quinolinic acid, where KMO, kynureninase and 3‐hydroxyanthranilic acid dioxygenase are involved. Several of the enzymes can be induced by immunological signals (bold lines).

Figure 1 depicts schematically the main steps of tryptophan‐kynurenine metabolism and demonstrates the supposed effects of immune activation found in this study.

**FIGURE 2:**
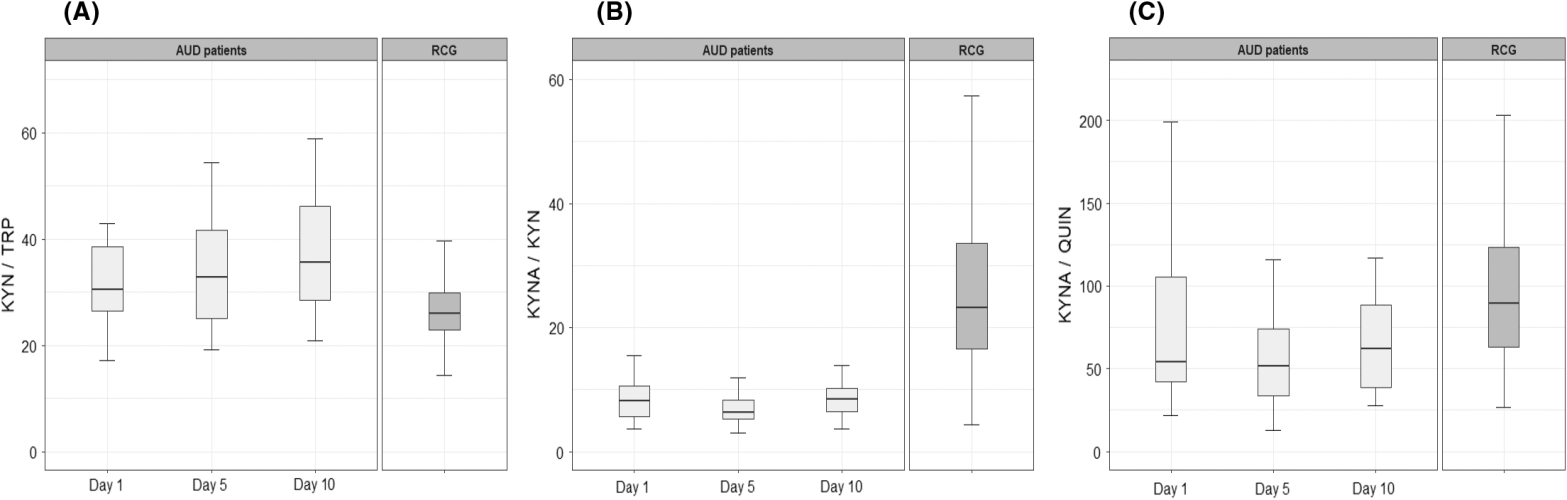
Parameters of kynurenine metabolism during alcohol withdrawal. KYN/TRP—kynurenine to tryptophan ratio, KYNA/KYN—kynurenic acid to kynurenine, KYNA/QUIN—kynurenic acid to quinolinic acid ratio, RCG—reference control group. (A) Boxplot presenting an increase of kynurenine to tryptophan ratio in AUD patients as compared to the RCG with the following statistical significance for Day 1: *p* = 0.002, Day 5: *p* = 0.003 and Day 10: *p* < 0.001. (B) Boxplot presenting a statistically significant decrease of kynurenic acid to kynurenine ratio in AUD patients as compared to the RCG (*p* < 0.001 for all three comparisons). (C) Boxplot presenting a decrease of kynurenic acid to quinolinic acid ratio in AUD patients as compared to the RCG (*p* < 0.001 for all three comparisons).

Table 1 presents concentrations of NEO, TRP, KYN and the KYN/TRP ratio as well as concentrations of KYNA and QUIN in AUD patients in comparison to reference values of reference control group (RCG; Arnhard et al., 2018; Geisler et al., 2015). Serum NEO concentrations were slightly higher in the AUD group as compared to RCG and increased between Day 1 and Day 10 of the study, but the observed differences did not achieve statistical significance (Table 1). The TRP levels were not significantly different between the groups in our sample and showed a slight non‐significant reduction by Day 10 of withdrawal. Serum KYN concentration was significantly increased in AUD patients as compared to the RCG (*p* < 0.05) at Day 1 and increased further through Days 5 and 10 (Table 1). KYN/TRP ratio was significantly higher in AUD patients as compared to RCG (Day 1: *p* = 0.002, Day 5: *p* = 0.003 and Day 10: *p* < 0.001, see also Figure 2).

**TABLE 1:** Concentrations of neopterin, tryptophan, kynurenine, quinolinic and kynurenic acid and group significances during acute alcohol withdrawal

	RCG mean (SD)	Day 1 mean (SD)	Day 5 mean (SD)	Day 10 mean (SD)
NEO (nM)	5.94 (1.57)	6.90 (5.33)	9.10 (10.79)	8.26 (5.07)
TRP (*μ*M)	67.4 (10.2)	65.6 (18.84)	64.6 (18.56)	61.2 (16.96)
KYN (*μ*M)	1.78 (0.42)	2.12 (0.64)*	2.29 (1.01)*	2.38 (0.95)**
KYN/TRP (*μ*M/*μ*M) × 1000	26.7 (6.2)	36.75 (20.47)**	38.27 (22.41)**	41.89 (24.62)***
QUIN (nM)	496 (240)	332 (296)***	328 (184)**	366 (235)*
KYNA (nM)	44.0 (18.2)	17.2 (12.3)***	16.1 (10.2)***	18.4 (10.3)***

*Note*: RCG—Reference control group, * significant *p* < 0.05; ** significant *p* < 0.01; ***significant *p* < 0.001; as compared to the RCG (Arnhard et al., 2018; Geisler et al., 2015); NEO, neopterin; TRP, tryptophan; KYN, kynurenine; KYN/TRP, kynurenine totryptophan ratio; QUIN, quinolinic acid; KYNA, kynurenic acid.

### Serum concentrations of quinolinic and kynurenic acid

Both serum QUIN and KYNA concentrations in this sample of AUD patients were lower than the concentration values of the RCG group, while KYNA showed much stronger reduction, down to 37% to 40% of the RCG values (see Table 1). As Figure 2B,C shows, the KYNA/KYN and KYNA/QUIN ratios were significantly lower in the AUD group in all three visits as compared to the RCG (*p* < 0.001 for both ratios).

### Correlations between NEO and parameters of tryptophan‐kynurenine metabolism

Table 2 shows that NEO concentrations and KYN/TRP ratio correlated significantly with QUIN concentrations (*r* = 0.646, *p* = 0.001, *r* = 0.555, *p* = 0.007, respectively) but not with KYNA concentrations (*r* = 0.392, *p* = 0.071, *r* = 0.276, *p* = 0.214, respectively). KYN concentrations correlated significantly and positively with KYN/TRP, QUIN and KYNA (*r* = 0.588, *p* = 0.004; *r* = 0.567, *p* = 0.006; *r* = 0.610, *p* = 0.003 respectively, Table 2).

**TABLE 2:** Correlations between neopterin, kynurenine/tryptophan ratio, kynurenine, kynurenic acid and quinolinic acid (mean concentration values over day 1, day 5 and day 10)

	NEO	KYN	KYN/TRP	QUIN
KYN	*r* = 0.407, *p* = 0.060			
KYN/TRP	*r* = 0.695, *p* < 0.001	*r* = 0.588, *p* = 0.004		
QUIN	*r* = 0.646, *p* = 0.001	*r* = 0.567, *p* = 0.006	*r* = 0.555, *p* = 0.007	
KYNA	*r* = 0.392, *p* = 0.071	*r* = 0.610, *p* = 0.003	*r* = 0.276, *p* = 0.214	*r* = 0.346, *p* = 0.115

Abbreviations: KYN/TRP, kynurenine‐to‐tryptophan ratio; KYNA, kynurenine acid; NEO, neopterin; QUIN, quinolinic acid.

In order to further elucidate the relationship between NEO, KYN, QUIN and KYNA linear mixed models analyses were performed with QUIN and KYNA as the dependent variables and NEO and KYN as the independent variables. Table 3 shows that both KYNA and QUIN concentrations were related positively and significantly to KYN concentrations. In contrast, only QUIN concentrations showed positive and significant association with NEO, while KYNA showed no significant association with NEO concentrations. A similar mixed models analysis involving NEO and KYN/TRP as independent variables demonstrated no significant associations between QUIN and KYNA concentrations and the KYN/TRP ratio.

**TABLE 3:** Estimates of fixed effects of neopterin (NEO) and kynurenine (KYN) on quinolinic acid (QUIN) and kynurenic acid (KYNA) metabolism with linear mixed models with AR(1) covariance structure

DV	IV	Estimate (β)	S.E.	df	*t*	*p*‐value	CI (95%)	
							LB	UB
Quinolinic acid (QUIN)	NEO	90.59	29.01	51.38	3.12	0.003	32.36	148.81
	KYN	105.21	30.87	31.46	3.41	0.002	42.29	168.13
Kynurenic acid (KYNA)	NEO	−0.80	1.58	55.07	−0.50	0.617	−3.97	2.38
	KYN	6.77	1.71	37.30	3.96	<0.001	3.30	10.23

Abbreviation: DV, dependent variable; IV, independent variable (predictor); Estimate (β), slope of the regression line; S.E., standard error; df, degrees of freedom; *t*‐value of the *t*‐statistics; CI, 95% confidence interval; LB, lower bound; UB, upper bound.

### Correlations between neuropsychiatric symptoms and KYN metabolites

Several items of the Obsessive–Compulsive Drinking Scale and Beck Depression Inventory correlated significantly with QUIN concentrations. The OCDS item Nr. 11 (“Anxiety and irritability”) correlated positively with QUIN concentrations (Spearman *r* = 0.433, *p* = 0.007), where the patients with higher concentrations of QUIN reported higher grades of a symptom. Similarly, the BDI items Nr. 15 (“Energy loss”) and Nr. 19 (“Concentration deficits”) showed a statistically significant positive correlation with QUIN concentrations (Spearman *r* = 0.370, *p* = 0.024 and *r* = 0.383, *p* = 0.019; respectively). The BDI item Nr. 19 (“Concentration deficits”) correlated negatively to the KYNA/QUIN ratio (Spearman *r* = −0.435, *p* = 0.007). NEO concentrations showed no correlations with neuropsychiatric symptoms.

## DISCUSSION

The main finding of this study is the significant increase of the KYN/TRP ratio in AUD patients during the alcohol withdrawal as compared to RCG and further increase of this ratio between the first and 10th days of the study. The increased activity of the KYN/TRP routes may imply less tryptophan availability for other metabolic routes such as serotonin formation. Simultaneously, we found a positive correlation between the immune activation marker NEO and KYN/TRP ratio, although NEO concentrations were marginally and not significantly increased. These findings support previous reports on moderately activated tryptophan to kynurenine metabolism during medium‐term alcohol withdrawal (Gleissenthall et al., 2014) and in AUD patients during alcohol withdrawal (Leclercq et al., 2021). Ample previous data have shown that this correlation is a reliable indicator of IDO‐1 activation by the immune system (Gostner et al., 2020; Murr et al., 2002), however a contribution of other isoenzymes cannot be completely excluded. Leclercq et al. (2021) suggest that hepatic TDO may be inhibited by active alcohol consumption and withdrawal could led to an enhancement of enzyme activity at least with prolonged abstinence (Leclercq et al., 2021). Thus, it is important to note that the increase of KYN/TRP may also be caused by a simultaneous activation of IDO and TDO enzymes. The AUD patients of our study showed only marginal immune activation as detected by CRP and NEO concentrations. Kyn concentrations were increased in AUD patients during alcohol withdrawal as compared to the reference values and increased further between day 1 and day 10, which is in agreement with the observations made by Leclercq et al. (2021) who describe a significant increase of KYN concentrations at the day 18 of alcohol withdrawal.

In this study, we found a significant correlation between serum NEO and serum QUIN concentrations. The relationship between immune activation and concentrations of the KYN metabolites QUIN and KYNA was additionally analyzed using linear mixed models, which allow to compare the impact of NEO concentrations and KYN on KYNA and QUIN concentrations, accounting for intra‐individual and inter‐individual levels of change. These analyses demonstrated that there was a significant effect of NEO and KYN on QUIN concentration, but KYNA levels were only influenced by KYN concentrations. This finding underlines the possibility that even a low‐grade immune activation may be associated with the enhanced activity of the metabolic route towards QUIN formation.

The kynurenine pathway involves several enzymes und leads to production of KYNA with involvement of kynurenine aminotransferases (KAT) on the one side and production of the 3‐hydroxykynurenine, and further metabolites including QUIN on the other side (see Figure 1). It is important to note that KAT enzymes are not inducible by cytokines, while kynureninase, kynurenine monooxygenase (KMO) and 3‐hydroxyanthranillic acid oxygenase can be induced by immune activation (Christmas et al., 2011; Myint & Kim, 2014). The intermediate metabolites; such as anthranilic acid, 3‐hydroxykynurenine and 3‐hydroxyanthranilic acid were not measured in this study. However, the significant correlation between NEO and serum QUIN concentrations, but not between NEO and KYNA, supports the suggestion that the immune‐induced activation of the kynurenine pathway in AUD may contribute to the imbalance of downstream metabolic routes. Thus, the processes leading to increased KYN catabolism may create a milieu in which the balance between different KYN metabolites is affected shifting the metabolic routes towards QUIN formation. The significant decrease of the KYNA/KYN and KYNA/QUIN as compared to the RCG found in this study support this suggestion. These findings are of particular interest because QUIN is able to exert glutamatergic (and potentially neurotoxic) effects, while KYNA possesses anti‐glutamatergic and neuroprotective properties (Christmas et al., 2011; Guillemin, 2012; Myint & Kim, 2014; Schwarcz & Du, 1991; Urbańska et al., 2014; Vécsei et al., 2013). Thus, changes of concentrations of these neuroactive metabolites may influence behavioral symptoms in AUD patients, as a recent study has demonstrated (Leclercq et al., 2021).

Patients with AUD, who try to abstain from alcohol, demonstrate a variety of neuropsychiatric symptoms long after the end the acute alcohol withdrawal. These protracted symptoms include depressive mood, anhedonia, anxiety, insomnia, energy loss and irritability seem to originate from the chronic alcohol‐associated glutamatergic hyperactivity (Burnett et al., 2016). Our data point towards a possible immune‐associated mechanism that may enhance glutamatergic activity in AUD patients by shifting the balance between different metabolites of the KYN pathway and thus potentially contribute to the risk of relapse. QUIN levels reported in this study for AUD patients during alcohol withdrawal were lower compared to values that were reported for control groups (Arnhard et al., 2018; Geisler et al., 2015), however levels increased with the time of withdrawal but remaining lower than control levels. We found some positive significant correlations between several items of the behavioral clinical rating scales and serum concentrations of QUIN and negative correlations with KYNA/QUIN ratio were observed for AUD patients during alcohol withdrawal. It is unclear, if these correlations are clinically relevant since we did not include medication‐free patients and measured only serum concentrations of metabolites. But some previous studies reported similar associations (Gleissenthall et al., 2014; Leclercq et al., 2021). It is also known that both QUIN and KYNA can be produced directly in the brain by different immune‐responsible cells (Melbourne et al., 2019; Vécsei et al., 2013). The immune‐associated activation of IDO has been investigated in various clinical conditions and has been shown to correlate with behavioral symptoms (Capuron et al., 2011; De Picker et al., 2020). The possible effects of the disturbed tryptophan‐kynurenine metabolism on behavioral symptoms during alcohol withdrawal remain largely unknown, although some recent studies have shown a potential association (Leclercq et al., 2021; Neupane et al., 2015; Vidal et al., 2020). It would be important for future research to understand how serum and cerebral fluid concentrations of KYN metabolites such as QUIN and KYNA interact with each other and how these interactions influence behavioral phenomena in alcohol withdrawal.

Increased degradation of TRP by the combined activity of the TDO (Badawy, 2013) and, as our study shows, by immune‐activated IDO may have important implications. First, it can lead to a decrease of TRP available for serotonin synthesis, thus affecting the serotoninergic system. It has been earlier suggested that this mechanism can contribute to the development of affective symptoms in different pathological conditions (Oxenkrug, 2010). Second, the immune‐associated activation of kynurenine pathway may cause a shift in kynurenine catabolism from a “neuroprotective arm” leading to production of KYNA towards the “neurotoxic arm” increasing production of 3‐hydroxykynureine and QUIN (Christmas et al., 2011, Myint & Kim, 2014; O'Farell & Harkin, 2017; Leclercq et al., 2021).

Our study has several important limitations. A small sample size of AUD patients restricts the possibilities for in‐depth analysis of different factors influencing interactions between immune activation, kynurenine metabolites and neuropsychiatric symptoms. As a proxy marker of immune activation we used neopterin which is an appropriate marker to detect cellular immune activation but we did not measured other immunological markers. All patients in this study were undergoing a symptom‐guided treatment with benzodiazepines. These treatment aims to alleviate alcohol withdrawal symptoms but do not generally affect other neuropsychiatric symptoms such as those documented by BDI and OCDS scales. Nevertheless, it could not be ruled out that some relevant neuro‐behavioral symptoms could have been masked by the effect of benzodiazepines. Furthermore, we did not measure the concentrations of the intermediate KYN metabolites such as anthranilic acid, 3‐hydroxykynurenine and 3‐hydroxyantranilic acid, which would give more detailed information on the disturbances of KYN metabolism. Moreover, it is not clear how long‐term alcohol consumption affects the enzymes involved in TRP and KYN downstream metabolism, as particular enzymes expressed in the liver are likely to be affected.

In sum, this study shows the immune‐associated activation of tryptophan‐kynurenine pathway in AUD patients during acute alcohol withdrawal and suggests that an assumed balance of concentrations of neuroactive KYN catabolites, such as QUIN and KYNA, may be specifically altered even by the low‐rate immune activation, also another mechanisms, such as hepatic metabolism may also play an important role. Further studies on the role of chronic immune activation in AUD should focus on the whole range of KYN metabolites and try to detect the possible long‐term effects of chronic immune activation in AUD on neurodegeneration and neuro‐behavioral symptoms. In particular, it would be important to investigate the effects of modulation of IDO‐1‐activity by medicaments and different nutritional factors in order to develop new approaches to the treatment of patients with AUD.

## CONFLICT OF INTEREST

The authors have no conflict of interest to declare.
